# Skin cancer excisions and histopathology outcomes when following a contemporary population‐based cohort longitudinally with 3D total‐body photography

**DOI:** 10.1002/ski2.216

**Published:** 2023-01-22

**Authors:** H. Peter Soyer, Montana O’Hara, Carina V. Silva, Caitlin Horsham, Dilki Jayasinghe, Saira Sanjida, Helmut Schaider, Joanne Aitken, Richard A. Sturm, Tarl Prow, Scott W. Menzies, Monika Janda

**Affiliations:** ^1^ Frazer Institute The University of Queensland Dermatology Research Centre Brisbane Queensland Australia; ^2^ Dermatology Department Princess Alexandra Hospital Brisbane Queensland Australia; ^3^ Faculty of Medicine Centre for Health Services Research The University of Queensland Brisbane Queensland Australia; ^4^ Cancer Council Queensland Brisbane Queensland Australia; ^5^ Institute for Resilient Regions University of Southern Queensland Brisbane Queensland Australia; ^6^ School of Public Health The University of Queensland Brisbane Queensland Australia; ^7^ Future Industries Institute University of South Australia Adelaide South Australia Australia; ^8^ Skin Research Centre York Biomedical Research Institute Hull York Medical School University of York York UK; ^9^ Sydney Melanoma Diagnostic Centre Royal Prince Alfred Hospital Camperdown New South Wales Australia; ^10^ Faculty of Medicine and Health The University of Sydney Camperdown New South Wales Australia

## Abstract

**Background:**

Skin cancer represents a significant health burden across the globe and early detection is critical to improve health outcomes. Three‐dimensional (3D) total‐body photography is a new and emerging technology which can support clinicians when they monitor people's skin over time.

**Objectives:**

The aim of this study was to improve our understanding of the epidemiology and natural history of melanocytic naevi in adults, and their relationship with melanoma and other skin cancers.

**Methods:**

Mind Your Moles was a 3‐year prospective, population‐based cohort study which ran from December 2016 to February 2020. Participants visited the Princess Alexandra Hospital every 6 months for 3 years to undergo both a clinical skin examination and 3D total‐body photography.

**Results:**

A total of 1213 skin screening imaging sessions were completed. Fifty‐six percent of participants (*n* = 108/193) received a referral to their own doctor for 250 lesions of concern, 101/108 (94%) for an excision/biopsy. Of those, 86 people (85%) visited their doctor and received an excision/biopsy for 138 lesions. Histopathology of these lesions found 39 non‐melanoma skin cancers (across 32 participants) and six in situ melanomas (across four participants).

**Conclusions:**

3D total‐body imaging results in diagnosis of a high number of keratinocyte cancers (KCs) and their precursors in the general population.

## Graphical abstract

Skin cancer represents a significant health burden across the globe and early detection is critical to improve health outcomes. Three‐dimensional (3D) total‐body photography is a new and emerging technology which can support clinicians when they monitor people's skin over time. This study provides data on the high number of keratinocyte cancers (KCs) and their precursors in the general population in Queensland. The results of this study inform the translation and implementation of 3D total‐body photography into clinical practice.

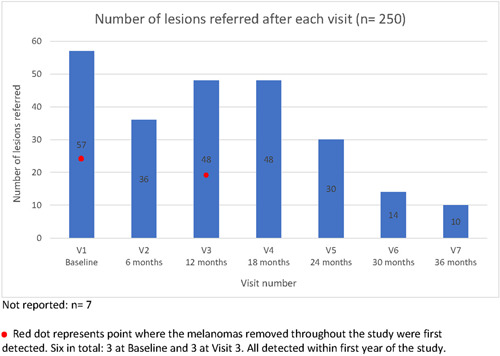

**What is already known about the topic?**

- Three‐dimensional (3D) total‐body imaging technology is currently used internationally, primarily in the research setting.

**What does this study add?**

- This study provides data on the high number of keratinocyte cancers (KCs) and their precursors in the general population in Queensland. The results of this study inform the translation and implementation of 3D total‐body photography into clinical practice.

## 1 INTRODUCTION

In 2020, an estimated 324 635 new cases of melanoma and 57 043 deaths occurred globally.^1^ Melanoma is the fourth most common cancer in Australia, with an estimated 28 000 new cases of in situ melanoma, 16 878 new cases of invasive melanoma and 1375 deaths in 2021.^2^, ^3^ Tumour thickness at diagnosis is the strongest predictor of survival. Patients diagnosed with thin melanomas (<0.8 mm) have a 10‐year survival rate of nearly 98%, compared to 55% for thickness of >4 mm.^4^ Keratinocyte cancers (KCs, also commonly called non‐melanoma skin cancers) are also a highly pressing health issue, with an estimated global incidence of 7.7 million cases in 2017 (65 000 deaths).^5^

Skin cancers are commonly diagnosed through skin examinations using a handheld dermatoscope. This can be complex and time consuming, as people have on average >30 naevi, as well as many other pigmented (e.g., seborrhoeic keratoses) and non‐pigmented lesions on their skin.^6^

Three‐dimensional (3D) total‐body photography is an emerging technology which allows for fast acquisition of high‐resolution macroscopic images of the skin surface, to support clinicians when they monitor people over time.^7^ Integrated software allows to sort lesions by characteristics such as size and colour and compare change over time. Dermoscopic images can be linked to the 3D images.

This longitudinal population‐based cohort study aimed to describe the clinical and histopathology outcomes of the Mind Your Moles study,^8^ a 3‐year prospective study of naevi in adults.

## 2 METHODS

This cohort study (for protocol see Koh et al.^8^) aimed to understand the natural history of melanocytic naevi in adults, and their relationship with melanoma and other skin cancers. The project enrolled adults living in Queensland, Australia, randomly selected from the Electoral Roll. Participants age 18–70 years, with Fitzpatrick skin type I–IV and at least one naevus were eligible. Participants underwent clinical skin examination and 3D total‐body photography using the VECTRA WB360 (Serial Number WB00009, Canfield Scientific, Parsippany, New Jersey, USA) every 6 months for 3 years.

The 92 camera 3D total‐body imaging machine simultaneously captures participant's whole skin surface, excluding areas covered by underwear, the hairy scalp and soles of feet, and then constructs a digital 3D avatar of the skin surface. Dermoscopic images of skin lesions the participant or study clinicians were concerned about, or pigmented lesions >5 mm were linked with their corresponding location and clinical image on the 3D avatar. The use of 3D total‐body photography allowed the study clinicians to compare images of the same lesion side by side over time, with identical angles and lighting.

### 2.1 Clinical skin examinations

During the study visits, any lesions suspicious for skin cancer identified by the study clinicians photographed using dermoscopy underwent tele‐review by a dermatologist (H. Peter Soyer) who determined if the lesion should be monitored, or immediately referred for management. No treatments were completed on the day of the study visit.

### 2.2 Referrals and histopathology

Participants with a lesion of concern were referred with a study letter to their own regular medical practitioner or the Hospital Dermatology Department. Letters of referral included anatomic description of lesion site, dermoscopic image and suspected/differential diagnosis provided by the dermatologist. Some were referred specifically for excision/biopsy/cryotherapy, others for monitoring. Histopathology reports were obtained from participant's treating doctor or pathology laboratory.

### 2.3 Data analyses

Sociodemographic and clinical participant characteristics, skin lesions referred, biopsied or excised and histopathological diagnoses were summarized using descriptive statistics.

In order to calculate the specificity and sensitivity of the clinical diagnoses, clinical and histopathology diagnoses (gold standard) were cross‐tabulated and standard formulae used.

### 2.4 Ethics

Ethics approval for this study was granted by the Metro South Health Human Research Ethics Committee (approval number: HREC/16/QPAH/125) and the University of Queensland Ethics Committee (approval number: 2016/HE000554).

## 3 RESULTS

### 3.1 Study visits

A total of 2100 participants were invited to participate, and recruitment ceased once 200 had accepted (see Supplementary Figure 1). Overall, 193 participants were enrolled at baseline and 164 participants remained at the final study visit (36 months), with 1213 (mean per participant 6.2 (range 1–7)) 3D‐imaging sessions completed between December 2016 and February 2020. At baseline, most participants were 50 years or older (*n* = 126/193, 65%) and 58% were male (*n* = 111/193). Most participants reported fair skin colour (*n* = 144/193, 75%); skin that burns then tans (*n* = 123/193, 64%); 13 (7%) reported they had been diagnosed with melanoma in the past and 48 (25%) a family history of melanoma (see Supplementary Table 1).

**FIGURE 1:**
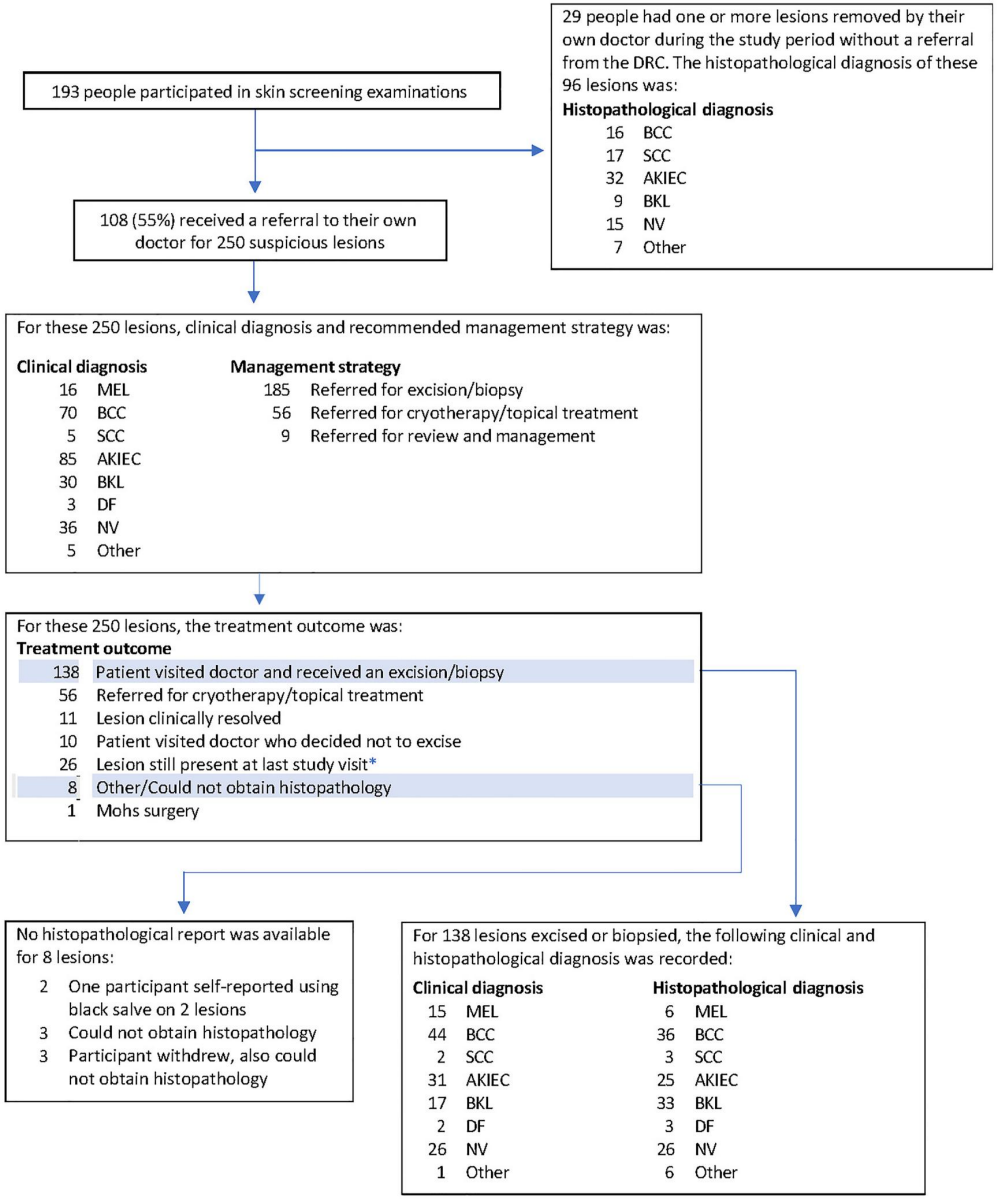
Clinical outcomes from the Mind Your Moles study. AKIEC, actinic keratosis (solar keratosis); intraepithelial carcinoma (Bowen disease); BCC, basal cell carcinoma; BKL, benign keratosis; actinic lentigo (solar lentigo); seborrhoeic keratosis; lichen‐planus like keratosis (lichenoid keratosis); DF, dermatofibroma; MEL, melanoma; NV, melanocytic naevus. SCC, squamous cell carcinoma. *Dermoscopic images of all 26 lesions not removed by the end of the study period were reviewed by an experienced dermatologist. No further action was required for 24/26 lesions. Of the remaining two lesions, one participant was recontacted to ensure a suspicious lesion was being monitored by their own dermatologist, and one participant was re‐sent referral letter to recommend excision.

**TABLE 1:** Clinical and histopathological diagnosis for lesions excised or biopsied (referred by the Dermatology Research Centre)

Histopathological diagnosis									
Clinical diagnosis^a^	Total (*n* = 138)	MEL (*n* = 6)	BCC (*n* = 36)	SCC (*n* = 3)	AKIEC (*n* = 25)	BKL (*n* = 33)	DF (*n* = 3)	NV (*n* = 26)	Other (*n* = 6)
MEL	15	6	1	‐	1	4	‐	3	‐
BCC	44	‐	29	1	2	6	‐	2	4^b^
SCC	2	‐	‐	‐	2	‐	‐	‐	‐
AKIEC	31	‐	5	2	17	7	‐	‐	‐
BKL	17	‐	‐	‐	2	13	‐	1	1^c^
DF	2	‐	‐	‐	‐	‐	1	‐	1^d^
NV	26	‐	1	‐	1	2	2	20	‐
Other^e^	1	‐	‐	‐	‐	1	‐	‐	‐

Abbreviations: AKIEC, actinic keratosis (solar keratosis); intraepithelial carcinoma (Bowen disease); BCC, basal cell carcinoma. BKL, benign keratosis; actinic lentigo (solar lentigo); seborrhoeic keratosis; lichen‐planus like keratosis (lichenoid keratosis); DF, dermatofibroma; MEL, melanoma; NV, melanocytic naevus; SCC, squamous cell carcinoma.

^a^ Clinical diagnosis refers to the diagnosis by the research dermatologist (H. Peter Soyer). Lesions clinically diagnosed as benign were excised to rule out a differential diagnosis, and/or because the participant was anxious/concerned about the lesion and wanted it removed.

^b^ *Other:* Warty dyskeratoma; tricholemmoma; hypertrophic scar; neurofibroma.

^c^ *Other:* Suppurative granulomatous inflammation.

^d^ *Other:* Prurigo nodule.

^e^ *Other:* Purpura which was identified as a new lesion and referred for biopsy. Histopathology found this lesion was an actinic lentigo.

### 3.2 Lesions referred from the clinical study visits

**FIGURE 2:**
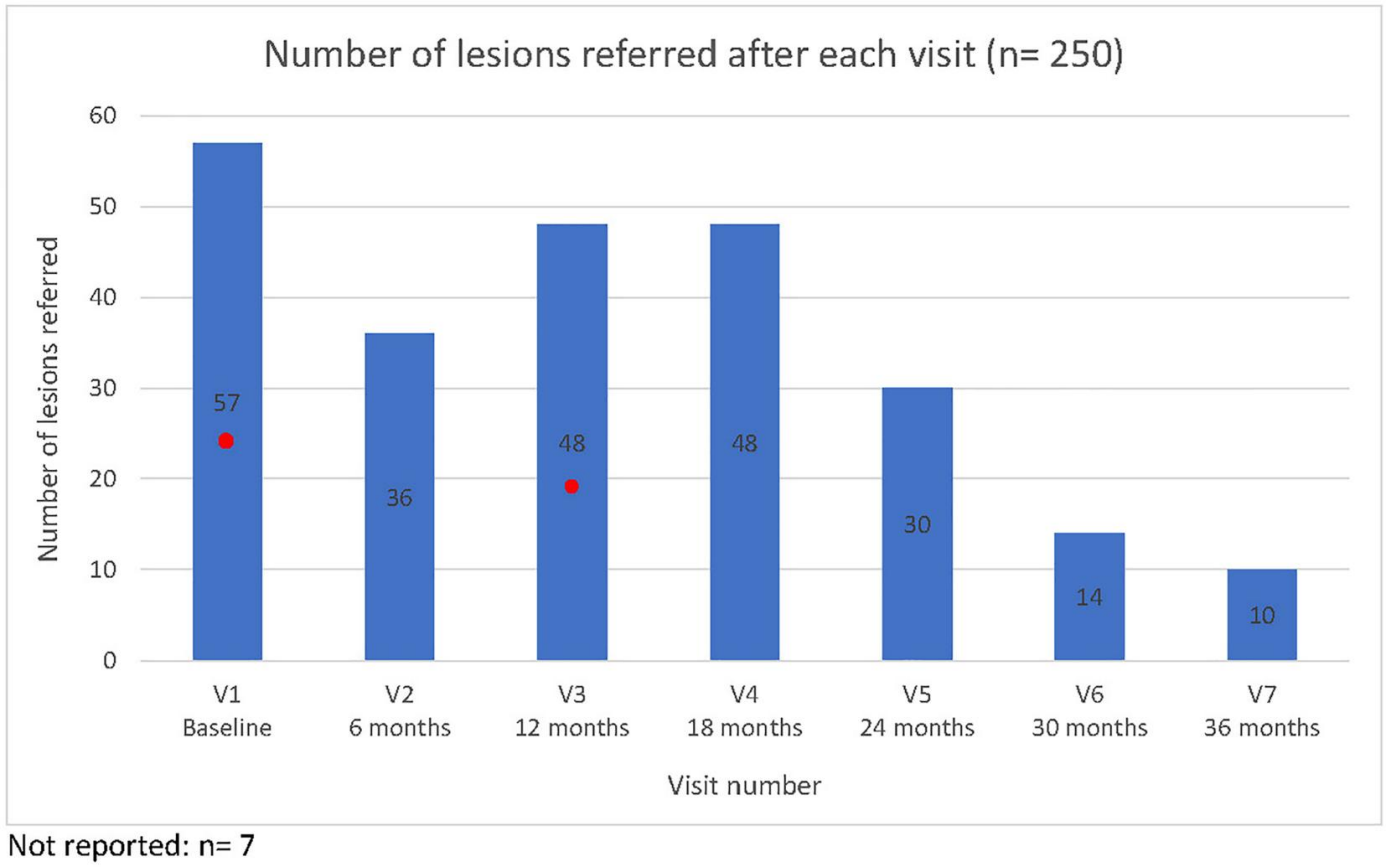
Number of lesions referred after each visit (*n* = 250). Red dot represents point where the melanomas removed throughout the study were first detected. Six in total: three at baseline and three at Visit 3. All detected within first year of the study.

**FIGURE 3:**
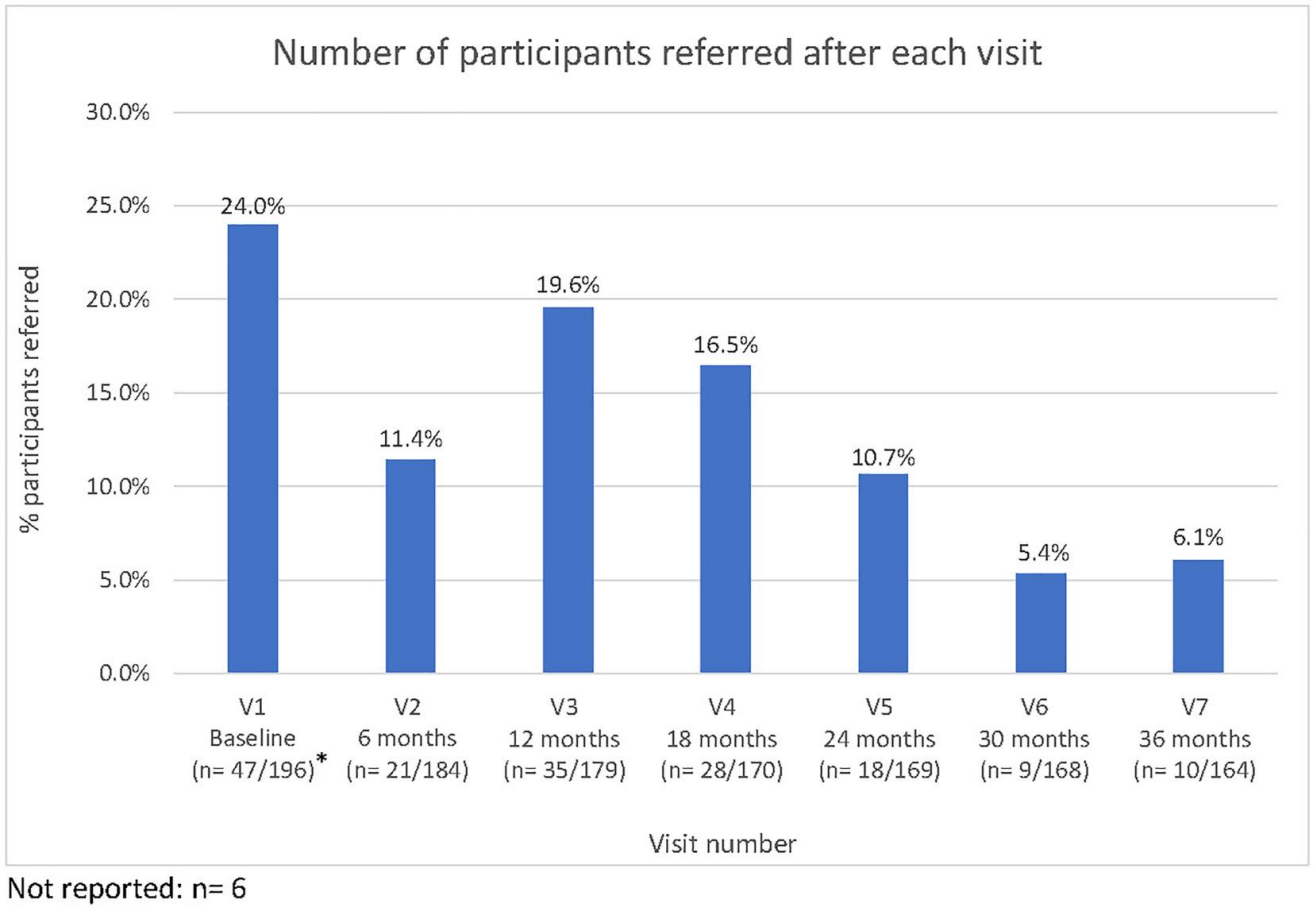
Number of participants referred after each visit. *Of the target sample of 196 participants who completed their baseline visit, one was excluded due to having a skin type outside the inclusion criteria, and two did not have 3D total‐body imaging.

Fifty‐six percent of participants (*n* = 108/193) received a referral for 250 lesions of concern (average 2.3 lesions per referral [range 1–9]). Of those, 63% were male (*n* = 68/108), and 75% were 50 years or older (*n* = 81/108); 9/109 (8%) had a personal history of melanoma; 26/108 (24%) had a family history. The recommended lesion management strategy was excision/biopsy (*n* = 185/250, 74%); cryotherapy/topical treatment (*n* = 56/250, 22%); or further review (*n* = 9/250, 4%) (Figure 1). The number of lesions referred was highest after Visit 1 (*n* = 57) and lowest after Visit 7 (36 months; *n* = 10; Figure 2). The proportion of participants referred after each visit was highest after Visit 1 (*n* = 47/196, 24.0%); lowest after Visit 6 (*n* = 9/168, 5.4%) (Figure 3).

Of 108 participant referrals, 101 (94%) recommended excision/biopsy. Of those, 86 participants (85%) visited their doctor and received excision/biopsy for 138 lesions. The outcomes for remaining 15 participants referred for excision/biopsy are described further below. A breakdown of the clinical and histopathology diagnosis of the 138 lesions excised/biopsied is provided in Table 1. Of the 138 lesions excised or biopsied, 61 were clinically suspicious to be skin cancer: 15 melanomas, 44 basal cell carcinoma (BCC) and 2 squamous cell carcinoma (SCC). The remaining lesions were suspected to be actinic keratosis, seborrhoeic keratosis or melanocytic naevi. In total, 36 participants were diagnosed with one or more skin cancers, 39 non‐melanoma skin cancers (across 32 participants) and 6 melanoma skin cancers (across four participants).

### 3.3 Remaining participants referred for excision/biopsy by the study

Of the remaining 15 participants who received a referral for excision/biopsy (*n* = 15/101), one had Mohs surgery (one lesion), and one visited their doctor who decided no excision necessary (two lesions). No record of attendance was received for the remaining 13 participants (17 lesions).

### 3.4 Melanomas detected by the study

Of the four participants (two males aged 43 and 56, and two females both age 64) with melanoma removed during the study, three had one melanoma, and one had three melanomas. All six were melanoma in situ, Clark Level 1, and half were combined with a dysplastic or compound naevus. They were excised from buttock (*n* = 1), shoulder (*n* = 2), chest (*n* = 2) and lower leg (*n* = 1). All six melanomas were diagnosed within the first 12 study months, three at baseline, three at visit 3 (12 months). The three melanomas detected at 12 months were photographed with dermoscopy and observed from baseline. One was initially thought to be seborrhoeic keratosis, later increased in pigmentation and was excised. The remaining two were thought to be dysplastic naevi (one asymmetric with multiple colours and a thick reticular network; and one which became enlarged with increased pigmentation). Both were excised to rule out melanoma in situ.

Of the remaining lesions clinically suspected to be melanoma (*n* = 9), histopathology was BCC, actinic lentigo, actinic keratosis (*n* = 1 each), seborrhoeic keratosis or melanocytic naevi (*n* = 3 each).

### 3.5 Sensitivity and specificity of clinical diagnosis

Melanoma, SCC and BCC were combined and counted as skin cancer. Of 138 excised/biopsied lesions, 61 were clinically suspicious for malignancy, of which 37 were histopathologically confirmed (true‐positives); 24 were benign (false‐positives). Of 77 lesions diagnosed as benign, 69 were histopathologically confirmed benign (true‐negative); 8 were malignant (false‐negative). Based on these, sensitivity and specificity of clinical diagnosis for skin cancer were 82% and 74%, respectively.

### 3.6 Lesions not referred from the study

Twenty‐nine participants had lesions removed by own doctor without referral from the study (*n* = 96 lesions). Of these, 26 had received a referral from the study for a different lesion, only three participants had no study referral. Histopathological outcomes of these 96 lesions are in Table 2.

**TABLE 2:** Histopathological diagnosis of lesions not referred by the Dermatology Research Centre (DRC)

			Histopathological diagnosis					
	Participants (*n* = 29)	Lesions (*n* = 96)	BCC (*n* = 16)	SCC (*n* = 17)	AKIEC (*n* = 32)	BKL (*n* = 9)	NV (*n* = 15)	Other^a^ (*n* = 7)
Participant received a referral from DRC for a different lesion	26	92	16	17	32	7	13	7
Participant did not receive a referral from DRC for any lesion	3	4	0	0	0	2	2	0

Abbreviations: AKIEC, actinic keratosis (solar keratosis); intraepithelial carcinoma (Bowen disease); BCC, basal cell carcinoma. BKL, benign keratosis; actinic lentigo (solar lentigo); seborrhoeic keratosis; lichen‐planus like keratosis (lichenoid keratosis); DF, dermatofibroma; MEL, melanoma; NV, melanocytic naevus; SCC, squamous cell carcinoma.

^a^ *Other:* Warty dyskeratoma; epidermal cyst; skin tag (*n* = 2); scar (*n* = 2); skin erosion (reactive epidermal hyperplasia).

### 3.7 Benign to malignant ratio

Over the course of the study, histopathology was received for 234 lesions. Of those, 72 were BCC or SCC and six melanomas. Of the 72 KCs, 39 were study referred and 33 were not, all six melanomas were study referred. The benign to malignant ratio for excised lesions in this study was 2.0:1.0 (156/78). The number needed to excise (NNE) was 3.0:1.0 (234/78).

## 4 DISCUSSION

This report summarises outcomes of a prospective population‐based cohort study when monitoring melanocytic naevi in adults over a 3‐year period using 3D total‐body photography.

The benefits of 2D total‐body photography for early detection of melanoma are well documented,^9^, ^10^ however this study is novel reporting outcomes from 3D total‐body photography and sequential digital dermoscopy imaging. The 3D total‐body imaging technology is currently used primarily in research settings and just sporadically in clinical settings, and this study can inform the translation and implementation of 3D total‐body photography in clinical practice.

Over the course of this study, just over half of participants received a referral to their own general practitioner or dermatologist, which is higher than reported in previous studies. For example, the community‐based melanoma screening programme by Aitken et al.^11^ with clinical examination by a general practitioner resulted in approximately 14% referrals. In a spot‐clinic screening in 2019, 100 biopsies were performed in 507 participants (19.7%).^12^ Participants were invited randomly from the Australian Electoral Roll, but this study's novel technology may have attracted individuals at increased skin cancer risk or already concerned about a skin lesion. This is consistent with demographic and phenotypic characteristics of our participants, with 25% family history of melanoma and 7% previous melanoma. The characteristics of participants in the present study differ to the study by Aitken et al.,^11^ where over half of the participants were younger than 50 years old (56%), and only 42% had fair skin.

Reflecting the high prevalence of skin cancers within this population, many referrals occurred at the baseline visit (*n* = 57). Of these referrals, 25% of lesions were found to be a skin cancer, including three melanomas. This may reflect the thoroughness of the assessment including 3D total‐body imaging, dermoscopy and clinical skin examination; or may be a result of the introduction of a new technology to which the clinicians were not yet used to. Previous research has explored the learning curve experienced by dermatologists following the adoption of dermoscopy, finding the benign to malignant ratio increased from 18.4:1 to 22.5:1 in the first year of dermoscopy use, but then decreased to 7.9:1 after approximately 18‐months^13^

Over time, 3D imaging may allow for identification of new, changing or stable naevi, supporting clinicians to detect even subtle changes which might have otherwise gone unnoticed while at the same time providing confidence for retaining stable lesions.^14^ Research has found that total‐body photography may allow to identify melanomas that do not have the classical clinical features.^15^ Change was the most important feature in most cases and it was also beneficial to avoid unnecessary biopsies.^15^

Although relatively non‐invasive compared to diagnostic tests for other cancers, having a skin biopsy has the potential for cost, cosmetic and patient anxiety consequences.^7^ The NNE is defined as the ratio of all lesions excised (including both benign and malignant) to the number of malignant lesions excised,^16^, ^17^ and was 3.0:1.0 (NNE 3) in this study. A prospective comparative study by Youl et al.^16^ found the NNE for a diagnosis of all skin cancer types combined was similar between general practitioners and skin cancer clinic doctors, at 2.1 and 1.9, respectively. The study by Baade et al.^17^ analysed the numbers of lesions excised for each skin cancer, finding the NNE was 1.5 for nonpigmented lesions (non‐melanoma skin cancers), and 19.6 for pigmented lesions (melanoma). The differences in the NNE in our study compared to these previous reports might have been related to differences in study setting or participants. The study by Baade et al.^17^ found that greater patient pressure as perceived by the clinicians was associated with higher number of excisions.

Clinical skin examinations and repeat total‐body photography often result in detecting in situ melanomas or melanoma with reduced thickness,^18^ compared to those detected by people themselves or presenting symptomatically.^11^, ^19^ In the present study, of the 138 lesions referred for excision, 15 were suspected to be melanoma, and six were histopathologically confirmed (4%). All six were melanoma in situ, highlighting the benefit of early detection. Three had been excised at baseline, but three were followed with dermoscopy for 12 months before excision due to the clinicians noticing changes in colour or size. In the future, 3D total‐body imaging will provide ample data on the number of lesions remaining stable over time or changing, and artificial intelligence algorithms can be developed to support clinician decision making, similar to those currently used in lung cancer screening programs.^20^

In our study, automated analysis software delivered skin lesions counts, and size, colour and border irregularity ratings. Further improvements of artificial intelligence algorithms are expected with many groups worldwide conducting research on the topic of clinical decision support,^21^ with some reporting excellent classification accuracy.^22^, ^23^, ^24^ Artificial intelligence development requires large datasets linked with clinical and histopathology data, and the present study can contribute to this effort.^25^ 3D imaging allows separate image acquisition and reporting, and has potential for teledermatology diagnosis which could improve access to dermatology diagnosis for people living in regional or remote areas, if the 3D total‐body photography was for example, integrated into public or private medical imaging departments.^7^

Limitations of the study include that despite the population‐based recruitment approach, participants may be biased towards those with interest in skin cancer early detection. The 96 lesions from 29 study participants not referred from the study should be counted as missed lesions. No specific reason could be identified for this, with the exception that all 29 study participants had severely photodamaged skin and lesions may have grown or become more evident in between 6‐monthly study visits. While the study followed participants thoroughly and retention over the 3 years was excellent (85%), some participants were lost to follow‐up.

## 5 CONCLUSION

This study shows that there is a high prevalence of melanoma, KCs and their precursors in the general population in Queensland. 3D total‐body imaging can play an increasingly important role in the early detection of skin cancer and has the potential to improve diagnostic accuracy and decrease overdiagnosis. Future research will compare the efficacy and cost–benefits of applying 2D and 3D total‐body photography and further evaluate the benefits of total‐body imaging and sequential digital dermoscopy imaging to inform its uptake in the clinical practice.

## CONFLICT OF INTEREST

H. Peter Soyer is a shareholder of MoleMap NZ Limited and e‐derm consult GmbH, and undertakes regular teledermatological reporting for both companies. H. Peter Soyer is a Medical Consultant for Canfield Scientific Inc., MoleMap Australia Pty Ltd., Blaze Bioscience Inc., Revenio Research Oy and a Medical Advisor for First Derm.

## Author contributions

**H. Peter Soyer:** Conceptualization (Equal); Funding acquisition (Lead); Methodology (Equal); Supervision (Equal); Writing – review and editing (Equal). **Montana O'Hara:** Data curation (Equal); Formal analysis (Equal); Writing – original draft (Equal); Writing – review and editing (Equal). **Carina V. Silva:** Formal analysis; Supporting, Writing – original draft (Equal). **Caitlin Horsham:** Writing – original draft (Equal); Writing – review and editing (Equal). **Dilki Jayasinghe:** Data curation (Equal); Formal analysis (Equal). **Saira Sanjida:** Writing – review and editing (Equal). **Helmut Schaider:** Methodology (Equal); Writing – review and editing (Equal). **Joanne Aitken:** Methodology (Equal); Writing – review and editing (Equal). **Richard A. Sturm:** Methodology (Equal); Writing – review and editing (Equal). **Tarl Prow:** Methodology (Equal); Writing – review and editing (Equal). **Scott W. Menzies:** Methodology (Equal); Writing – review and editing (Equal). **Monika Janda:** Conceptualization (Equal); Methodology (Equal); Supervision (Equal); Writing – review and editing (Equal).

## ETHICS STATEMENT

Ethics approval for this study was granted by the Metro South Health Human Research Ethics Committee (approval number: HREC/16/QPAH/125) and the University of Queensland Ethics Committee (approval number: 2016/HE000554).

## Supporting information

Supplementary Material 1

Supplementary Material 2

## Data Availability

The data that support the findings of this study are available from the corresponding author upon reasonable request. The data are not publicly available due to privacy or ethical restrictions.
