# Supplementary material for: Skin cancer excisions and histopathology outcomes when following a contemporary population‐based cohort longitudinally with 3D total‐body photography
Source: Skin Health Dis. 2023 Jan 22;3(2):e216. doi: 10.1002/ski2.216 (PMC10066755; doi:10.1002/ski2.216)
Supplement: Supplementary file 1 — Supplementary Material 1 [file SKI2-3-e216-s001.docx]

**Figure S1.** Participant flow diagram

Contact details received from AEC (n=5000)

Not required (n=2900)

Randomised and sent invitation (n=2100)

Declined invitation (n=81)

No response (n=1814)

Interested and screened for eligibility (n=205)

Ineligible (n=1)

Participant enrolled (n=204)

Withdrew (n=8)

Completed baseline visit (n=196)*

Withdrew (n=12)

Completed 6-month visit (n=184)

Withdrew (n=5)

Completed 12-month visit (n=179)

Withdrew (n=9)

Completed 18-month visit (n=170)

Withdrew (n=1)

Completed 24-month visit (n=169)

Withdrew (n=1)

Completed 30-month visit (n=168)

Withdrew (n=4)

Completed 36-month visit
(n=164)

* Of the target sample of 196 participants who completed their baseline visit, one was excluded due to having a skin type outside the inclusion criteria, and 2 did not have 3D total-body imaging.

*AEC:* Australian Electoral Commission
