# Supplementary material for: Skin cancer excisions and histopathology outcomes when following a contemporary population‐based cohort longitudinally with 3D total‐body photography
Source: Skin Health Dis. 2023 Jan 22;3(2):e216. doi: 10.1002/ski2.216 (PMC10066755; doi:10.1002/ski2.216)
Supplement: Supplementary file 2 — Supplementary Material 2 [file SKI2-3-e216-s002.docx]

**Table S1.** Baseline characteristics of 193 participants

| **Characteristic** | **n (%)** |
| --- | --- |
| **Sex** |  |
| Female | 82 (42) |
| Male | 111 (58) |
| **Age** |  |
| 20-29 | 9 (5) |
| 30-39 | 27 (14) |
| 40-49 | 31 (16) |
| 50-59 | 63 (33) |
| 60-69 | 63 (33) |
| **Highest education Level** |  |
| High school or less | 46 (24) |
| Post school qualification | 147 (76) |
| **Innate skin colour** |  |
| Fair | 144 (75) |
| Medium | 48 (25) |
| Olive | 1 (1) |
| **Skin reaction to acute sun** |  |
| Only tan | 18 (10) |
| Burn then tan | 123 (64) |
| Always burn | 52 (27) |
| **Painful/peeling sunburns before age 20** |  |
| ≤10 | 76 (39) |
| 10+ | 117 (61) |
| **Occupational sun exposure** |  |
| Mainly outdoors | 80 (41) |
| Both indoors and outdoors/ mainly indoors | 113 (59) |
| **Leisure sun exposure** |  |
| Mainly outdoors | 167 (87) |
| Both indoors and outdoors/ mainly indoors | 26 (13) |
| **Family history of melanoma** |  |
| No | 144 (75) |
| Yes | 49 (25) |
| **Personal history of melanoma** |  |
| No | 180 (93) |
| Yes | 13 (7) |
| **Past keratinocyte cancer** |  |
| No | 139 (72) |
| Yes | 54 (28) |
